# Renal involvement in paediatric inflammatory bowel disease

**DOI:** 10.1007/s00467-019-04413-5

**Published:** 2019-12-09

**Authors:** Mohamed Mutalib

**Affiliations:** Department of Paediatric Gastroenterology, Evelina London Children’ Hospital, Westminster Bridge Road, London, SE1 7EH UK

**Keywords:** Paediatric IBD, Renal disease in IBD, Inflammatory bowel disease, Kidneys and IBD, Extraintestinal manifestation IBD

## Abstract

Inflammatory bowel disease (IBD), which includes Crohn’s disease, ulcerative colitis and inflammatory bowel disease unclassified, is a chronic inflammatory disorder that predominantly affects the gastrointestinal (GI) tract and has a rising incidence in both children and adults. Symptoms are caused by inappropriate inflammatory response triggered by interaction between the environment, gut microbiome and host immune system in a genetically susceptible individual. Extranintestinal manifestations of IBD are common and can affect any body system outside the gut; they can precede or run parallel to GI inflammation. Renal involvement in IBD is uncommon and can be part of extraintestinal manifestation or metabolic complications of IBD. Many medications used to treat IBD can cause renal damage. Renal manifestation in children with IBD can range from asymptomatic biochemical abnormalities to variable stages of renal impairment with significant morbidity and even mortality burden.

## Introduction

Inflammatory bowel disease (IBD) is a chronic, relapsing and remitting inflammatory condition predominantly affecting the gastrointestinal (GI) tract that results from complex interplay of the individual’s genetic makeup, the environment, the gut microbiota and the host immune response [1]. IBD is an umbrella disorder that covers Crohn’s disease, ulcerative colitis and IBD unclassified [2]. Crohn’s disease is characterised by transmural inflammation that can affect any part of the GI tract with characteristic skip lesions of healthy tissues, while ulcerative colitis is a continuous mucosal inflammation largely confined to the colon. ‘IBD unclassified’ is a term used when the endoscopic and histological features are not sufficient to favour one of the other diagnoses [2].

Extraintestinal manifestations of IBD are disorders linked to IBD and can affect virtually any body system outside the GI tract [3]. While some of the affected organs can have a clearly defined inflammatory drive, such as pyoderma gangrenosum in the skin, ankylosing spondyolitis in the musculoskeletal system and sclerosing cholangitis in the liver, the inflammation can occur either as a sequelae of GI inflammation or can follow a parallel independent course to IBD [4]. Other symptoms known to be affected by IBD complications, such as osteoporosis, venous thrombosis, gallbladder and kidney stones are better regarded as extraintestinal complications rather than manifestations of IBD [5]. Medications used to treat IBD can also cause symptoms independent of IBD inflammation or complications.

Extraintestinal manifestation of IBD is common and is reported in about 25–40% of cases in both adults and children, while renal involvement in IBD is less prevalent and ranges from 4–23% in adults and 1–2% in children with IBD [6, 7]. Interestingly, extraintestinal symptoms can precede the first presentation of IBD or can occur after the surgical resection of the affected bowel, or can also develop simultaneously with gastrointestinal symptoms. Although multifactorial in origin, the extraintestinal manifestation of IBD in general, and renal involvement in particular, can be explained by one of the following mechanisms:

- IBD activity-independent: IBD-independent autoimmune disorder that reflects host susceptibility to autoimmunity based on common genetic predisposition and disorganised immune system. This can lead to autoimmune-mediated renal dysfunction [5]. Renal disease in this category is directly related to luminal gastrointestinal inflammation.
- IBD activity-dependent: Reactive manifestation associated with IBD disease activity and bowel inflammation [8].
- Deposition of, or local formation of, immune complexes in the target organ [9]^.^
- Metabolic abnormalities secondary to malabsorption or as a consequence of intestinal surgery [7].

Medication adverse effects [6] are considered a separate entity under complications, rather than extraintestinal manifestations.

Most of the renal manifestations of IBD are reported in case reports or relatively small case series, but the common types of renal disorders in children with IBD can be grouped under:

- Metabolic consequences, e.g. calculi
- Inflammatory conditions, e.g. tubulonephritis and glomerulonephritis
- Amyloidosis
- Medication adverse effects

## Metabolic renal conditions

### Nephrolithiasis

Nephrolithiasis is uncommon in children, but the incidence of renal stones in adult patients with IBD is higher than that reported in the general population, with an estimated range of 12–28% in IBD patients compared to 5% in the general population [10–12]. They are more prevalent in Crohn’s disease than ulcerative colitis and are predominantly uric acid or calcium oxalate in composition [13]. The cause of oxalate stones in patients with IBD is complex, but mainly stems from malabsorption of bile salts and fatty acids, both predominantly absorbed in the terminal ileum, which leads to increase in intestinal oxalate absorption [14]. As most dietary oxalate is bound to calcium and is poorly absorbed, fat malabsorption secondary to inflamed or resected terminal ileum will deliver an abundance of fat in the intestinal lumen to bind calcium, thus increasing free oxalate which is readily absorbed to the blood stream via the GI mucosa [15]. Periods of dehydration and reduced urine volume are not uncommon in IBD patients and are invariably coupled with reduced urinary magnesium and citrate (both are regarded as stone inhibitors), which will increase the susceptibility of developing oxalate stones [16].

Uric acid stones on the other hand develop in the context of increased GI bicarbonate and fluid loss leading to concentrated and acidic urine, which may enhance the precipitation of renal uric acid in the state of reduced concentration of stone inhibitors [17]. Interestingly, IBD patients with uric acid stones do not have increased blood concentration or urinary excretion of uric acid, rendering these unsuitable as monitoring markers [7]. Children with IBD can also develop calcium phosphate stones through a poorly understood mechanism [11]. Renal stones can also occur as an adverse effect of IBD medications.

Nephrolithiasis treatment should be directed towards reversing the metabolic abnormalities, such as increasing fluid intake in patients with excessive GI losses to guard against stoma losses and diarrhoea, alkalinisation of urine and citrate administration [10]. Low-fat diet and bile salt sequestrants can help in cases of steatorrhea or bile salt malabsorption, respectively [18].

### Tubular injury and renal insufficiency

Although drugs used to treat IBD can cause renal impairment, renal insufficiency and tubular injury can be an independent extraintestinal manifestations of IBD, with reported prevalence between 2 and 16% of cases [19]. Using proteinuria and enzymuria as markers for renal tubular damage, it is clear that both markers are elevated in a subset of patients with IBD and are strongly correlated with active IBD [20]. This tubular damage appears to recover with appropriate IBD therapy, removing medication adverse effects as a cause [21]. Asymptomatic haematuria and proteinuria with normal renal function are also common in patients with IBD and are more frequent in Crohn’s disease than ulcerative colitis [22].

## Inflammatory conditions

### Glomerulonephritis

The pathophysiology of glomerulopathy in patients with IBD is complex and poorly understood [6], but the correlation of glomerulonephritis with intestinal inflammation and the improvement of renal disease with control of IBD inflammation suggests that glomerulonephritis is mostly an extraintestinal manifestation rather than a consequence of inflammatory or metabolic disruption [5, 7, 23].

The most common type of glomerulonephritis in IBD is IgA nephropathy, either primary or secondary type [24–26]. IBD and idiopathic IgA nephropathy share a common genetic linkage and an association with HLA-DR1 is found in both conditions [27–29]. Considering the relatively high incidence of subclinical IgA nephropathy in the general population [30], some authors argue the association with IBD is coincidental; however, over the last few decades, an increasing level of evidence supports a true association with clinical and histological remission of renal disease achieved with IBD treatment [7].

Other types of glomerulonephritis including membranoproliferative glomerulonephritis, minimal change disease, membranous glomerulopathy, antiglomerular basement membrane glomerulonephritis and C3 glomerulopathy have been described in case reports and small case series.

Different mechanisms are implicated in the development of glomerulonephritis in IBD, including cytokine-induced inflammation leading to both glomerular and GI inflammation and damage [31]. IgG4 may also plays a role through direct deposition of IgG4 complexes in the glomeruli inducing inflammation or through antigen cross-reactions [32]. Mucosal break and loss of surface integrity secondary to GI inflammation can lead to increased exposure to antigens and a response increase in immunoglobulin levels. The ensuing immune complexes can subsequently be deposited in the glomeruli [6].

Treatment is aimed at controlling the IBD inflammation, which will usually result in improvement and on occasion normalisation of renal function. Although follow-up renal biopsy to document histological remission of glomerulonephritis is not a recognised practice, the biochemical improvement or renal function would suggest histological healing.

### Tubulointerstitial nephritis

Tubulointerstitial nephritis is the second most common renal disorder affecting children with IBD [33, 34]. Although most cases are secondary to medication adverse effects, which will be discussed in detailed later on this review, in some cases, tubulointerstitial nephritis is diagnosed simultaneously with IBD prior to initiation of any therapy and is related to IBD activity, pointing towards an extraintestinal disorder [20].

Proximal tubular dysfunction presenting as proteinuria is described in up to 6% of patients with IBD [22]. Tubular specific marker proteins, including alpha one and beta two microglobulin are low molecular weight proteins that are usually filtered by glomeruli and reabsorbed by the proximal tubules providing sensitive markers for tubular damage [35]. Granulomatous tubulointerstitial inflammation observed in renal biopsies from patients with Crohn’s disease further support the link to IBD rather than as an effect of medications [9].

Secondary tubulointerstitial nephritis can result from hypokalaemia (due to GI losses, particularly in patients with stomas and extensive bowel surgery), hyperoxaluria and renal amyloidosis. Such a diverse aetiology may explain the discrepancy in treatment outcome: some authors have reported improvement in renal function with optimisation of IBD treatment, while others have found no such correlation [36]. Treatment should simultaneously address intestinal inflammation as well as correcting nephrotoxic metabolic predisposition.

## Amyloidosis

Renal amyloidosis is a rare but serious complication of IBD, and is associated with significant morbidity and even mortality burden [37]. Secondary amyloidosis, the predominant type of amyloidosis in patients with IBD, is characterised by extracellular deposition of serum amyloid A, an acute phase reactant protein in different parts of the kidneys, leading to proteinuria or renal insufficiency [38]. Deposition of amyloid in the glomeruli will usually lead to proteinuria and/or nephrotic syndrome, while interstitial amyloid accumulation will result in renal insufficiency [39].

Secondary amyloidosis is more prevalent in Crohn’s disease compared to ulcerative colitis, with reported incidence of up to 10.9% in Crohn’s disease and 0.7% in ulcerative colitis [23]. It can take between 10 and 15 years after IBD diagnosis to develop secondary amyloidosis; however, in rare instances, it can manifest with the initial presentation of IBD [7]; as such, it is mainly an adult disorder but a few cases in paediatric patients with IBD have been described [33, 40].

The aim of treatment of secondary amyloidosis is focused on reducing the level of serum amyloid A to minimise end organ deposition. In the context of IBD, this is usually achieved by controlling the intestinal inflammation, and steroid and immune modulators have been tried with variable level of success [41]. Infliximab (an anti-TNF alpha) appears to produce IBD remission and reduce proteinuria from secondary amyloidosis, and while it does not reverse the renal damage as measured by serum creatinine, it seems to stop the progression of chronic kidney disease [42].

## Drug-related nephrotoxicity

Most medications used in the treatment of IBD are known to have nephrotoxic adverse effects, although at times it may not be possible to differentiate renal damage caused by extraintestinal manifestation from drug nephrotoxic action.

### Aminosalicylates

5-aminosalicylates (5-ASA) are commonly used for colonic inflammation in both ulcerative colitis and Crohn’s disease to achieve local anti-inflammatory action at the inflamed part of the intestine [43]. As the free 5-ASA is unstable in acid and is readily absorbed in the proximal small intestine, several mechanisms have been developed to carry the therapeutic ingredient to the colon. In sulphasalazine, the 5-ASA moiety is bound to sulfapyridine; in osalazine, it is linked to another 5-ASA and is attached to an inert carrier in balsalazide; and in pentasa, asacol and salofalk, the 5-ASA is coated with an acid-resistant coating which allows release in the alkaline colon [44].

Subclinical renal injury is reported in as high as 1 in every 100 adult patients taking 5-ASA, but clinically significant impairment is thought to happen in about 1 in 500. However, there is no robust paediatric epidemiological data, as most of the literature is in isolated case reports and small case series [45, 46].

Renal damage secondary to 5-ASA is usually in the form of tubulointerstitial nephritis, but glomerulonephritis and minimal change nephropathy with nephrotic syndrome have also been reported [47]. It is not precisely known how 5-ASA causes renal damage but different theories have been proposed. Analgesic nephropathy is a common cause of drug-induced renal disease characterised by papillary necrosis and interstitial nephritis, usually caused by non-steroidal anti-inflammatory drugs. 5-ASA shares structural similarities to salicylates and can lead to hypoxic renal damage either by uncoupling oxidative phosphorylation in renal mitochondria or by inhibiting the synthesis of renal prostaglandins, exacerbating tissue hypoxia and reperfusion damage. Dose-related, idiosyncratic effects have been reported, particularly with sulphasalazine, which may be part of a generalised hypersensitivity reaction. On the other hand, several clinical trials have concluded that dosage or duration of exposure are not factors in mesalazine-induced interstitial nephritis [7, 48].

Early detection of renal damage, caused either by idiosyncratic effects or nephrotoxic actions of 5-ASA and the immediate cessation of the offending drug, may lead to reversal of the renal injury. However, when diagnosis is delayed, the effect on the kidneys can be long-lasting and steroids or immune modulators may be required to improve renal function [49].

Regular monitoring of renal function is strongly recommended during 5-ASA therapy. Although, no universally agreed monitoring protocol has been developed. Some authors suggesting measuring renal function before starting 5-ASA, monthly for 3 months, 3 monthly for 9 months and 6 monthly thereafter. Special attention and frequent monitoring should be considered in patients receiving steroids, as these may mask symptoms of renal damage [7, 8].

### Azathioprine and mercaptopurine

Thiopurines (azathioprine and mercaptopurine) are the standard first-line immune modulator therapy for maintenance of remission in IBD [43]. They are extensively used and widely tolerated. Case reports of interstitial nephritis in non-IBD patients treated with azathioprine have been reported. Nephrotoxicity is also considered as an adverse effect of thiopurines; hence, monitoring of renal function while on azathioprine therapy is advisable [50, 51].

### Tumour necrosis factor alpha (TNFα) inhibitors

Anti TNFα treatments are commonly used in IBD for both induction of remission and maintenance therapy for children and adults with moderate to severe IBD. They are frequently used in isolation or combined with other immune modulators such as azathioprine [52]. Infliximab (a chimeric anti-TNF IgG1 monoclonal antibody) and adalimumab (a recombinant anti-TNF IgG1 monoclonal antibody) are the most commonly used anti TNFα [52]. Anecdotal observations and case reports suggest clinical benefits from using anti TNFα in the treatment of glomerulonephritis in IBD patients. Furthermore, anti TNFα can be effective in the treatment of renal amyloidosis in patients with IBD. However, anti TNFα can contribute to renal injury and may directly cause glomerulonephritis or lupus nephritis [53, 54].

One of the well-documented effects of using anti TNFα in general, and infliximab in particular, is the development of autoantibodies which may lead to loss of efficacy. As it is known that glomerular visceral epithelial cells can produce TNFα [55], the interaction between TNFα and anti TNFα antibodies (produced as a consequence of infliximab therapy) can result in glomerulonephritis and membranous nephropathy. Infliximab as an anti TNFα can also directly bind glomerular produced TNFα leading to cell death and apoptosis [56]. It is not uncommon during treatment with anti TNFα to develop anti-nuclear antibodies, anti-dsDNA antibodies and anti-neutrophil cytoplasmic antibodies, which may lead to lupus-like and/or crescentic glomerulonephritis in some patients [5].

Apart from a single case report of an adult patient who developed lupus nephritis after using golimumab (a new anti TNFα), there is no data on the renal effects of golimumab [57].

### Tacrolimus

Tacrolimus is a macrolide antibiotic that inhibits T-lymphocyte signal transduction and IL-2 transcription. It is not one of the standard immune modulators used in IBD, but it has been sparingly used especially in cases of refractory ulcerative colitis [58]. Similar to cyclosporine, tacrolimus can cause acute and chronic nephrotoxic effects. Acute kidney injury is caused by a reversible vasoconstriction of the afferent arterioles leading to reduction blood flow, while chronic kidney damage is caused by irreversible interstitial fibrosis [59].

Elevated serum creatinine is one of the common renal adverse effects of tacrolimus use—an increase of up to 30% from baseline has been described. However, data on long-term nephrotoxic effects are conflicting, likely representing the small number of patients in all published studies. Mild elevation of serum creatinine with short-term (less than 4 months) tacrolimus use has been reported [60, 61].

### Vedolizumab

Vedolizumab is a relatively new biologic therapy for moderate to severe IBD. It acts by blocking the binding of integrin-expressing cells to MAdCAM (mucosal addressin cell adhesion molecule) on the surface of mucosal endothelial cells [62]**.** Vedolizumab was initially thought to be renal friendly, but at least one published case reports an acute interstitial nephritis secondary to vedolizumab. The renal injury recovered completely with systemic steroids and the authors were able to reintroduce Vedolizumab with pre- and post-administration steroid cover with no further effects on renal function [63].

## Conclusion

Renal involvement in children with IBD can be secondary to extraintestinal manifestations or complication of IBD. It can present with a wide range of renal disorders and can affect the glomeruli and/or the tubular structure. Most medications used to treat IBD have nephrotoxic properties which may not be related to dose or duration of therapy. It is important to recognise the early signs of renal damage and to investigate all possible causative factors to maximise the chances of halting or reversing function deterioration.

## Summary points

- Renal involvement can be part of extraintestinal manifestations or complications of IBD.
- Most medications used to treat IBD can cause renal damage.
- Renal manifestation of IBD can range from asymptomatic proteinuria and haematuria to renal insufficiency.
- Renal disease can precede the intestinal inflammation or it can occur after surgical resection of the affected bowel
- Early identification of contributing factors will help guide treatment and may reverse the deterioration in renal function.

## Multiple choice questions (answers are provided after the references)

1. Renal disease as extraintestinal manifestation of IBD can be caused by:
  - Genetic predisposition to immune deficiency disorders.
  - Medications adverse effects.
  - Deposition of immune complexes in the kidneys.
  - Crohn’s disease more than ulcerative colitis.
  - Duration of intestinal inflammation is a leading factor.
2. In an adolescent with an oxalate renal stone secondary to IBD:
  - The likely cause is GI bicarbonate loss and acidic urine.
  - Low-fat diet and bile salt chelating agents can prevent recurrence.
  - Alkalinisation of urine can prevent recurrence.
  - Calcium phosphate stones are more common that oxalate stones.
  - Renal stones are very common in children with IBD.
3. A child with Crohn’s disease well controlled on azathioprine for the last 3 years developed glomerulonephritis and renal insufficiency:
  - IgA nephropathy is the most common type in children with IBD.
  - Stopping azathioprine is recommended.
  - Long-term outcome in children is better than in adults.
  - HLA DQ2 and DQ8 are common in IgA nephropathy and IBD.
  - Renal replacement therapy should be considered early.
4. Mesalazine is commonly used in children with IBD and can lead to renal impairment:
  - Drug dose and duration of therapy are major factors in mesalazine nephrotoxic effect.
  - Concomitant steroid use can increase the risk of renal damage.
  - Immune modulators can improve intestinal inflammation and renal function.
  - 2 monthly blood test is routinely recommended.
  - Concomitant use of azathioprine at IBD diagnosis increases the risk of renal damage.
5. Infliximab (an anti TNFα) is recommended for treatment of moderate to severe cases of IBD:
  - Prolonged use of infliximab can worsen renal amyloidosis.
  - Anti-infliximab antibodies can cause direct glomerular damage.
  - Renal stones are common during treatment with infliximab.
  - Renal tubules can produce TNF receptors.
  - Infliximab can cause reversible renal vasoconstriction.
